# Supplementary material for: Evolution of Quorum Sensing in Pseudomonas aeruginosa Can Occur via Loss of Function and Regulon Modulation
Source: mSystems. 2022 Oct 3;7(5):e00354-22. doi: 10.1128/msystems.00354-22 (PMC9600717; doi:10.1128/msystems.00354-22)
Supplement: TABLE S1 [file msystems.00354-22-s0003.docx]

| **Clone** | **Group** | **Gene** | **Description** | **Mutation** | **Reference** | **Variant** | **Start position of mutation** | **Stop position of mutation** | **Size or position of mutation** | **Position on the gene** | **Amino acid residue** |
| --- | --- | --- | --- | --- | --- | --- | --- | --- | --- | --- | --- |
| 1 | Las | *lasI-rsaL-lasR* | Las signal, repressor, and receptor | Big Deletion |  |  | 1540028 | 1565202 | 25174 |  |  |
| 2 | Las | *lasI-rsaL-lasR* | Las signal, repressor, and receptor | Big Deletion |  |  | 1538124 | 1566949 | 28825 |  |  |
| 3 | Las | *lasI-rsaL-lasR* | Las signal, repressor, and receptor | Big Deletion |  |  | 1541540 | 1563604 | 22064 |  |  |
| 4 | Las | *lasI-rsaL-lasR* | Las signal, repressor, and receptor | Big Deletion |  |  | 1498290 | 1562211 | 63921 |  |  |
| 5 | Las | *lasI-rsaL-lasR* | Las signal, repressor, and receptor | Big Deletion |  |  | 1498291 | 1562211 | 63920 |  |  |
| 6 | Las | *lasI-rsaL-lasR* | Las signal, repressor, and receptor | Big Deletion |  |  | 1558737 | 1563640 | 4903 |  |  |
| 7 | Las | *lasI-rsaL-lasR* | Las signal, repressor, and receptor | Big Deletion |  |  | 1555339 | 1565763 | 10424 |  |  |
| 8 | Las | *lasI-rsaL-lasR* | Las signal, repressor, and receptor | Big Deletion |  |  | 1555455 | 1569517 | 14062 |  |  |
| 9 | Las | *lasI-rsaL-lasR* | Las signal, repressor, and receptor | Big Deletion |  |  | 1555455 | 1569516 | 14061 |  |  |
| 10 | Las | *lasI-rsaL-lasR* | Las signal, repressor, and receptor | Big Deletion |  |  | 1496833 | 1562802 | 65969 |  |  |
| 11 | Las | *lasI-rsaL-lasR* | Las signal, repressor, and receptor | Big Deletion |  |  | 1531740 | 1569561 | 37821 |  |  |
| 12 | Las | *lasI-rsaL-lasR* | Las signal, repressor, and receptor | Big Deletion |  |  | 1496833 | 1562801 | 65968 |  |  |
| 13 | Las | *lasI-rsaL-lasR* | Las signal, repressor, and receptor | Big Deletion |  |  | 1553890 | 1563643 | 9753 |  |  |
| 14 | Las | *lasI-rsaL-lasR* | Las signal, repressor, and receptor | Big Deletion |  |  | 1533017 | 1564452 | 31435 |  |  |
| 15 | Las | *lasI-rsaL-lasR* | Las signal, repressor, and receptor | Big Deletion |  |  | 1544760 | 1562460 | 17700 |  |  |
| 16 | Las | *lasI-rsaL-lasR* | Las signal, repressor, and receptor | Big Deletion |  |  | 1556546 | 1568383 | 11837 |  |  |
| 17 | Las | *lasI-rsaL-lasR* | Las signal, repressor, and receptor | Big Deletion |  |  | 1556547 | 1568384 | 11837 |  |  |
| 18 | Las | *lasI-rsaL-lasR* | Las signal, repressor, and receptor | Big Deletion |  |  | 1539213 | 1568281 | 29068 |  |  |
| 19 | Las | *lasI-rsaL-lasR* | Las signal, repressor, and receptor | Big Deletion |  |  | 1557905 | 1564000 | 6095 |  |  |
| 20 | Las | *lasI-rsaL-lasR* | Las signal, repressor, and receptor | Big Deletion |  |  | 1514042 | 1566917 | 52875 |  |  |
| 21 | Las | *lasI-rsaL-lasR* | Las signal, repressor, and receptor | Big Deletion |  |  | 1531740 | 1569561 | 37821 |  |  |
| 22 | Las | *lasI-rsaL-lasR* | Las signal, repressor, and receptor | Big Deletion |  |  | 1544367 | 1567581 | 23214 |  |  |
| 23 | Las | *lasI-rsaL-lasR* | Las signal, repressor, and receptor | Big Deletion |  |  | 1544366 | 1567581 | 23215 |  |  |
| 24 | Las | *lasI-rsaL-lasR* | Las signal, repressor, and receptor | Big Deletion |  |  | 1542363 | 1569062 | 26699 |  |  |
| 25 | Las | *lasI-rsaL-lasR* | Las signal, repressor, and receptor | Big Deletion |  |  | 1542140 | 1563698 | 21558 |  |  |
| 26 | Las | *lasI-rsaL-lasR* | Las signal, repressor, and receptor | Big Deletion |  |  | 1554842 | 1566542 | 11700 |  |  |
| 27 | Las | *lasI-rsaL-lasR* | Las signal, repressor, and receptor | Big Deletion |  |  | 1554842 | 1566543 | 11701 |  |  |
| **Clone** | **Group** | **Gene** | **Description** | **Mutation** | **Reference** | **Variant** | **Start position of mutation** | **Stop position of mutation** | **Size or position of mutation** | **Position on the gene** | **Amino acid residue** |
| 28 | Las | *lasR* | Las receptor | SNP | T | G | 1558837 | 1558837 | 1558837 | 669 | 223 |
| 29 | Las | *lasR* | Las receptor | SNP | T | G | 1558837 | 1558837 | 1558837 | 669 | 223 |
| 30 | Las | *lasR* | Las receptor | SNP | C | T | 1558840 | 1558840 | 1558840 | 672 | 224 |
| 31 | Las | *lasR* | Las receptor | SNP | C | T | 1558840 | 1558840 | 1558840 | 672 | 224 |
| 32 | Las | *lasR* | Las receptor | SNP | C | T | 1558840 | 1558840 | 1558840 | 672 | 224 |
| 33 | Rhl | *rhlR* | Rhl receptor | SNP | A | G | 3890367 | 3890367 | 3890367 | 285 | 95 |
| 34 | Rhl | *rhlR* | Rhl receptor | SNP | A | G | 3890367 | 3890367 | 3890367 | 285 | 95 |
| 35 | Rhl | *rhlR* | Rhl receptor | SNP | G | A | 3890407 | 3890407 | 3890407 | 246 | 82 |
| 36 | PQS | *pqsR* | PQS receptor | SNP | A | G | 1086473 | 1086473 | 1086473 | 60 | 20 |
|  |  | *pqsB* | PQS signal operon | INDEL | AGGGG | AGGG | 1080061 | 1080061 | 1080061 | 624 | 208 |
| 37 | PQS | *pqsR* | PQS receptor | SNP | C | T | 1086399 | 1086399 | 1086399 | 699 | 233 |
|  |  | *pqsC* | PQS signal operon | SNP | A | G | 1081318 | 1081318 | 1081318 | 468 | 156 |
| 38 | PQS | *pqsD* | PQS signal operon | SNP | C | A | 1082220 | 1082220 | 1082220 | 279 | 93 |
| 39 | PQS | *pqsE* | PQS signal operon | SNP | G | A | 1082951 | 1082951 | 1082951 | 3 | 1 |
| 40 | PQS | *pqsE* | PQS signal operon | SNP | G | A | 1082951 | 1082951 | 1082951 | 3 | 1 |
| 41 | PQS | *pqsE* | PQS signal operon | SNP | T | C | 1083111 | 1083111 | 1083111 | 165 | 55 |
| 42 | PQS | *pqsE* | PQS signal operon | SNP | T | C | 1083111 | 1083111 | 1083111 | 165 | 55 |
| 43 | PQS | *pqsR* | PQS receptor | SNP | A | G | 1086806 | 1086806 | 1086806 | 291 | 97 |
| 44 | PQS | *pqsR* | PQS receptor | SNP | A | G | 1086806 | 1086806 | 1086806 | 291 | 97 |
| 45 | PQS | *pqsR* | PQS receptor | SNP | A | G | 1086987 | 1086987 | 1086987 | 111 | 37 |
| 46 | PQS | *pqsR* | PQS receptor | INDEL | GTT | GT | 1086354 | 1086355 | 1086354 | 744 | 248 |
| 47 | PQS | *pqsR* | PQS receptor | SNP | A | G | 1086980 | 1086980 | 1086980 | 117 | 39 |
| 48 | PQS | *pqsR* | PQS receptor | INDEL | ACC | AC | 1086298 | 1086299 | 1086296 | 801 | 267 |
| 49 | PQS | *pqsR* | PQS receptor | INDEL | TGCTGACCGCCGAGCTGACCGC | TGCTGACCGC | 1086985 | 1086997 | 1086991 | 105 | 34 - 37 |
| 50 | PQS | *pqsR* | PQS receptor | INDEL | TGCTGACCGCCGAGCTGACCGC | TGCTGACCGC | 1086985 | 1086997 | 1086991 | 105 | 34 - 37 |
| 51 | PQS | *pqsR* | PQS receptor | SNP | C | T | 1086399 | 1086399 | 1086399 | 699 | 233 |
| 52 | PQS | *pqsR* | PQS receptor | SNP | C | T | 1086311 | 1086311 | 1086311 | 786 | 262 |
| **Clone** | **Group** | **Gene** | **Description** | **Mutation** | **Reference** | **Variant** | **Start position of mutation** | **Stop position of mutation** | **Size or position of mutation** | **Position on the gene** | **Amino acid residue** |
| 53 | PQS | *pqsR* | PQS receptor | SNP | T | G | 1086919 | 1086919 | 1086919 | 177 | 59 |
| 54 | PQS | *pqsR* | PQS receptor | INDEL | TGCTGACCGCCGAGCTGACCGC | TGCTGACCGC | 1086 985 | 1086997 | 1086991 | 105 | 34 - 37 |
| 55 | PQS | *pqsR* | PQS receptor | INDEL | TGCTGACCGCCGAGCTGACCGC | TGCTGACCGC | 1086985 | 1086997 | 1086991 | 105 | 34 - 37 |
| 56 | PQS | *pqsR* | PQS receptor | INDEL | CGGG | CGG | 1086568 | 1086569 | 1086568 | 531 | 177 |
| 57 | Las + PQS | *lasI-rsaL-lasR* | Las signal, repressor, and receptor | Big Deletion |  |  | 1557989 | 1566221 | 8232 |  |  |
|  | Las + PQS | *pqsR* | PQS receptor | SNP | C | A | 1086366 | 1086366 | 1086366 | 732 | 244 |
| 58 | Las + PQS | *lasI-rsaL-lasR* | Las signal, repressor, and receptor | Big Deletion |  |  | 1540616 | 1565563 | 24947 |  |  |
|  | Las + PQS | *pqsR* | PQS receptor | SNP | G | T | 1086289 | 1086289 | 1086289 | 807 | 269 |
| 59 | Las + PQS | *lasR* | Las receptor | SNP | G | T | 1558213 | 1558213 | 1558213 | 45 | 15 |
|  | Las + PQS | *pqsR* | PQS receptor | INDEL | TGCTGACCGCCGAGCTGACCGC | TGCTGACCGC | 1086985 | 1086997 | 1086991 | 105 | 34 - 37 |
| 60 | Rhl + PQS | *rhlR* | Rhl receptor | SNP | T | C | 3890139 | 3890139 | 3890139 | 513 | 171 |
|  | Rhl + PQS | *pqsR* | PQS receptor | SNP | A | G | 1087034 | 1087034 | 1087034 | 63 | 21 |
| 61 | Rhl + PQS | *rhlR* | Rhl receptor | SNP | A | T | 3889938 | 3889938 | 3889938 | 714 | 238 |
|  | Rhl + PQS | *pqsR* | PQS receptor | SNP | C | T | 1087093 | 1087093 | 1087093 | 3 | 1 |
