# Supplementary material for: Evolution of Quorum Sensing in Pseudomonas aeruginosa Can Occur via Loss of Function and Regulon Modulation
Source: mSystems. 2022 Oct 3;7(5):e00354-22. doi: 10.1128/msystems.00354-22 (PMC9600717; doi:10.1128/msystems.00354-22)
Supplement: TABLE S2 [file msystems.00354-22-s0004.docx]

|  | **Low Iron** | **Mid Iron** | **High Iron** |
| --- | --- | --- | --- |
| **Low Viscosity** | 4 | 7 | 7 |
| **Mid Viscosity** | 8 | 10 | 16 |
| **High Viscosity** | 4 | 8 | 7 |
