# Supplementary material for: Evolution of Quorum Sensing in Pseudomonas aeruginosa Can Occur via Loss of Function and Regulon Modulation
Source: mSystems. 2022 Oct 3;7(5):e00354-22. doi: 10.1128/msystems.00354-22 (PMC9600717; doi:10.1128/msystems.00354-22)
Supplement: TABLE S3 [file msystems.00354-22-s0005.docx]

| **Clone** | **Average coverage of *aphA*** | **Standard deviation** |
| --- | --- | --- |
| 1 | 187.2 | 17.5 |
| *2 | 0 | 0 |
| *3 | 0 | 0 |
| 4 | 197.4 | 16.2 |
| 5 | 166.2 | 18.2 |
| 6 | 214.0 | 21.0 |
| 7 | 157.1 | 15.0 |
| 8 | 159.5 | 12.9 |
| 9 | 199.2 | 13.9 |
| 10 | 170.8 | 13.2 |
| 11 | 161.8 | 18.3 |
| *12 | 0 | 0 |
| *13 | 0 | 0 |
| 14 | 222.0 | 20.6 |
| 15 | 195.6 | 17.6 |
| 16 | 232.6 | 23.0 |
| 17 | 175.3 | 18.7 |
| 18 | 184.4 | 10.7 |
| *19 | 0 | 0 |
| *20 | 0 | 0 |
| 21 | 187.0 | 14.6 |
| *22 | 0 | 0 |
| 23 | 288.2 | 12.1 |
| 24 | 260.1 | 20.9 |
| 25 | 299.3 | 22.9 |
| 26 | 246.1 | 26.0 |
| 27 | 218.7 | 12.7 |
| 28 | 278.6 | 21.2 |
| *29 | 0 | 0 |
| WT PAO1 | 254.3 | 28.7 |
