# Supplementary material for: Evolution of Quorum Sensing in Pseudomonas aeruginosa Can Occur via Loss of Function and Regulon Modulation
Source: mSystems. 2022 Oct 3;7(5):e00354-22. doi: 10.1128/msystems.00354-22 (PMC9600717; doi:10.1128/msystems.00354-22)
Supplement: TABLE S4 [file msystems.00354-22-s0006.docx]

|  | **PC1** | **PC2** | **PC3** | **PC4** | **PC5** |
| --- | --- | --- | --- | --- | --- |
| **Growth** | *0.3557* | -0.3695 | 0.7588 | 0.2855 | 0.2823 |
| **Protease** | -0.2117 | *0.5752* | 0.5797 | -0.5369 | 0.0043 |
| **Pyocyanin** | -0.2236 | *-0.6773* | -0.0374 | -0.6764 | 0.1800 |
| **Rhamnolipid** | *-0.5975* | -0.2593 | 0.2930 | 0.2690 | -0.6462 |
| **Biofilm** | *-0.6494* | 0.0819 | -0.0301 | 0.3168 | 0.6858 |
| **Explained variance (%)** | 32.9 | 24.6 | 18.3 | 14.7 | 9.5 |
