# Supplementary material for: Evolution of Quorum Sensing in Pseudomonas aeruginosa Can Occur via Loss of Function and Regulon Modulation
Source: mSystems. 2022 Oct 3;7(5):e00354-22. doi: 10.1128/msystems.00354-22 (PMC9600717; doi:10.1128/msystems.00354-22)
Supplement: TABLE S5 [file msystems.00354-22-s0007.docx]

| **Strains** | **Description** | **Source or reference** |
| --- | --- | --- |
| PAO1 wild type *promoterless-GFP and mCherry* | Non-fluorescent wild type strain with an empty promoter site fused to GFP and mCherry | P. Jayakumar, S. A. Thomas, S. P. Brown, and R. Kümmerli, bioRxiv, 2021, https://doi.org/10.1101/2021.03.22.436499 |
| **Evolved *rhlR* mutants** | | |
| Clone 33-*lasR-gfp-rhlR-mCherry* | Transcriptional fusion of *lasR-GFP* and *rhlR*-*mCherry* from pDR05 | This study |
| Clone 33-*lasB-gfp-rhlA-mCherry* | Transcriptional fusion *lasB-GFP* and *rhlA*-*mCherry* from pDR06 | This study |
| Clone 34-*lasR-gfp-rhlR-mCherry* | Transcriptional fusion of *lasR-GFP* and *rhlR*-*mCherry* from pDR05 | This study |
| Clone 34-*lasB-gfp-rhlA-mCherry* | Transcriptional fusion *lasB-GFP* and *rhlA*-*mCherry* from pDR06 | This study |
| Clone 35-*lasR-gfp-rhlR-mCherry* | Transcriptional fusion of *lasR-GFP* and *rhlR*-*mCherry* from pDR05 | This study |
| Clone 35-*lasB-gfp-rhlA-mCherry* | Transcriptional fusion *lasB-GFP* and *rhlA*-*mCherry* from pDR06 | This study |
| **Evolved *rhlR* mutants containing mutations in *pqsR*** | | |
| Clone 60-*lasR-gfp-rhlR-mCherry* | Transcriptional fusion of *lasR-GFP* and *rhlR*-*mCherry* from pDR05 | This study |
| Clone 60-*lasB-gfp-rhlA-mCherry* | Transcriptional fusion *lasB-GFP* and *rhlA*-*mCherry* from pDR06 | This study |
| Clone 61-*lasR-gfp-rhlR-mCherry* | Transcriptional fusion of *lasR-GFP* and *rhlR*-*mCherry* from pDR05 | This study |
| Clone 61-*lasB-gfp-rhlA-mCherry* | Transcriptional fusion *lasB-GFP* and *rhlA*-*mCherry* from pDR06 | This study |
