# Supplementary material for: Evolution of Quorum Sensing in Pseudomonas aeruginosa Can Occur via Loss of Function and Regulon Modulation
Source: mSystems. 2022 Oct 3;7(5):e00354-22. doi: 10.1128/msystems.00354-22 (PMC9600717; doi:10.1128/msystems.00354-22)
Supplement: TABLE S6 [file msystems.00354-22-s0008.docx]

|  | **PC1** | **PC2** | **PC3** | **PC4** | **PC5** |
| --- | --- | --- | --- | --- | --- |
| **Growth** | 0.3119 | *-0.6463* | 0.3768 | 0.0073 | 0.5856 |
| **Protease** | *-0.4887* | -0.3860 | 0.1128 | -0.7395 | -0.2291 |
| **Pyocyanin** | *0.5054* | 0.3571 | -0.2834 | -0.6613 | 0.3153 |
| **Rhamnolipid** | 0.2635 | 0.3355 | 0.8406 | -0.1252 | -0.3094 |
| **Biofilm** | *-0.5822* | *0.4396* | 0.2416 | -0.0061 | 0.6398 |
| **Explained variance (%)** | 34.1 | 30.5 | 19.4 | 12.6 | 3.5 |
