# Supplementary material for: Evolution of Quorum Sensing in Pseudomonas aeruginosa Can Occur via Loss of Function and Regulon Modulation
Source: mSystems. 2022 Oct 3;7(5):e00354-22. doi: 10.1128/msystems.00354-22 (PMC9600717; doi:10.1128/msystems.00354-22)
Supplement: TABLE S7 [file msystems.00354-22-s0009.docx]

| **Clone** | **Protease production** | **Protein Domain** | **Mutation type** |
| --- | --- | --- | --- |
| 36 | Low | DBD | Frameshift |
| 43 | Low | LBD | Missense |
| 44 | Low | LBD | Missense |
| 45 | Low | DBD | Missense |
| 47 | Low | DBD | Missense |
| 48 | Low | LBD | Frameshift |
| 49 | Low | DBD | Conservative in-frame deletion |
| 52 | Low | LBD | Missense |
| 53 | Low | DBD | Missense |
| 56 | Low | LBD | Frameshift |
| 37 | High | DBD | Missense |
| 46 | High | DBD | Frameshift |
| 50 | High | LBD | Conservative in-frame deletion |
| 51 | High | DBD | Missense |
| 54 | High | LBD | Conservative in-frame deletion |
| 55 | High | LBD | Conservative in-frame deletion |
