# Supplementary material for: Evolution of Quorum Sensing in Pseudomonas aeruginosa Can Occur via Loss of Function and Regulon Modulation
Source: mSystems. 2022 Oct 3;7(5):e00354-22. doi: 10.1128/msystems.00354-22 (PMC9600717; doi:10.1128/msystems.00354-22)
Supplement: TABLE S8 [file msystems.00354-22-s0010.docx]

| **Strains** | **Description** | **Source or reference** |
| --- | --- | --- |
| ***E. coli*** | | |
| CC118 λpir | Δ (*ara, leu*)_7697_ *araD*139 *ΔlacX74 galE galK phoA20 thi-1 rpsE rpoB* (Rf^R^) *argE(am) recA1 λpir^+^* | V. De Lorenzo, M. Herrero, U. Jakubzik, and K. N. Timmis, J Bacteriol 172:6568–6572, 1990,  https://doi.org/10.1128/jb.172.11.6568-6572.1990 |
| ***Pseudomonas aeruginosa*** | | |
| PAO1 wild type | Wild type strain (ATCC 15692) | This laboratory |
| PAO1 *ΔlasR* | Deficient in the receptor of Las system | This laboratory |
| PAO1 *ΔrhlR* | Deficient in the receptor of Rhl system | This laboratory |
| PAO1 *ΔlasR* *ΔrhlR* | Deficient in the receptor of both Las and Rhl systems | This laboratory |
| **Plasmids** | | |
| pUX-BF13 | Helper plasmid to provide Tn7 transposase proteins for reporter construct integration at the *att*Tn7 site in *P. aeruginosa* | Y. Bao, D. P. Lies, H. Fu, G. P. Roberts, Gene 109:167–168, 1991,  https://doi.org/10.1016/0378-1119(91)90604-a |
| pDR05 | pUC18-mini-Tn7-Gm with *lasR-GFP* and *rhlR-mCherry* | P. Jayakumar, S. A. Thomas, S. P. Brown, and R. Kümmerli, bioRxiv, 2021, https://doi.org/10.1101/2021.03.22.436499 |
| pDR06 | pUC18-mini-Tn7-Gm with *lasB-GFP* and *rhlA-mCherry* | P. Jayakumar, S. A. Thomas, S. P. Brown, and R. Kümmerli, bioRxiv, 2021, https://doi.org/10.1101/2021.03.22.436499 |
